# Draft Genome Sequence of *Chryseobacterium mucoviscidosis* sp. nov. Strain VT16-26, Isolated from the Bronchoalveolar Lavage Fluid of a Patient with Cystic Fibrosis

**DOI:** 10.1128/genomeA.01473-17

**Published:** 2018-01-11

**Authors:** George Tetz, Victor Tetz

**Affiliations:** a Human Microbiology Institute, New York, New York, USA; b TGV-Biomed, New York, New York, USA

## Abstract

We report here the draft genome sequence of *Chryseobacterium mucoviscidosis* VT16-26, a novel bacterium isolated from the lungs of a patient with cystic fibrosis. The genome was composed of 4,403,956 bp and had 36.2% G+C content. We detected 4,048 genes with predicted protein-coding functions, including those associated with antibiotic resistance and virulence.

## GENOME ANNOUNCEMENT

The *Chryseobacterium* genus is composed of Gram-negative, aerobic, nonmotile, and rod-shaped bacteria. The *Chryseobacterium* species are uncommon human pathogens, predominantly implicated in infections in immunocompromised hospitalized patients with severe underlying diseases (1–3). However, members of the *Chryseobacterium* family have never been associated with cystic fibrosis. Using combined culture and genetic workflow, we have previously identified the unexplored diversity of bacteria within the human lungs (4–6).

The 16S rRNA gene of *Chryseobacterium mucoviscidosis* VT16-26, which was isolated from the bronchoalveolar lavage fluid of a patient with cystic fibrosis, was found to possess ≤98% sequence identity with other representatives of *Chryseobacterium* species. The genome of *Chryseobacterium mucoviscidosis* VT16-26 was sequenced using the HiSeq 2500 (GA IIx; Illumina, CA), according to the manufacturer’s instructions. A draft genome was assembled using SPAdes (version 3.5.0), with 156-fold average coverage (7).

In total, 255 assembled contigs, which had 4,403,956 bp and a 36.2% G+C content, were annotated using the NCBI Prokaryotic Genome Annotation Pipeline (8). The genome harbors 83 tRNA genes, 7 rRNA and 3 noncoding RNA (ncRNA) operons, and 4,048 protein-coding sequences. We identified the presence of genes conferring resistance to antibiotics, such as vancomycin, bleomycin, teicoplanin, and tetracycline, via its major facilitator superfamily (MFS) efflux pump, as well as multidrug resistance transporters of the ABC, multiantimicrobial extrusion (MATE), and MFS families. Virulence factors, such as hemolysin D, metalloproteases, peptidases, deoxyribonucleases, ribonucleases, alpha-amylase, and adhesins, were identified in the genome.

In comparison with the genome of its closest relative, *Chryseobacterium gambrini* DSM 18014, the genome of *Chryseobacterium mucoviscidosis* VT16-26 is smaller (4,841,687 bp versus 4,403,956 bp). An *in silico* DNA-DNA hybridization (DDH) analysis confirmed that the genomes of *Chryseobacterium gambrini* DSM 18014 and *Chryseobacterium mucoviscidosis* VT16-26 belonged to two different species. This was inferred because the Genome-to-Genome Distance Calculator algorithm produced a DDH value of 58.90%, which was well below the threshold value of 70% (9, 10).

Follow-up studies of *Chryseobacterium mucoviscidosis* VT16-26 and its harbored bacteriophages would enable us to understand its possible pathogenicity and role in cystic fibrosis (11, 12).

### Accession number(s).

The complete genome sequence has been deposited in the NCBI database under accession no. MVAG00000000.
